# Characterization of breast changes in the early gestational period on automated breast ultrasound

**DOI:** 10.1007/s10396-023-01370-3

**Published:** 2023-10-24

**Authors:** Tomoyuki Ohta

**Affiliations:** Radiology Department, International University of Health and Welfare Hospital, 537-3 Iguchi, Nasushiobara City, Tochigi 329-2763 Japan

**Keywords:** Ultrasonography, Breast, Pregnant, Gestational period, Milk duct

## Abstract

**Purpose:**

This study was conducted to determine the characteristics of milk duct development in early pregnancy on ultrasound images.

**Methods:**

Automated breast ultrasound (ABUS) images used for breast cancer screening in 332 pregnant women were evaluated retrospectively to determine when and how ductal development becomes evident on ultrasonography. The diagnostic criteria used for mammary gland changes during the gestational period were extension of the ducts to the margins of the breast where little or no echogenic fibroglandular tissue is seen on sonograms and/or the appearance of ductal structures running along the ascending Cooper’s ligament tapering off or ending in a blind end at the superficial layer of the superficial fascia. The correlations between gestational stage and the prevalence of these criteria were verified by Spearman’s rank correlation coefficient (*ρ*). Assessments were performed by a single radiologist with experience reading ABUS images.

**Results:**

With a few exceptions, the prevalence of the above findings increased sharply beginning at 10 weeks, and then increased with progression of gestation, reaching a plateau after 20 weeks (*ρ* = 0.766, *P* < 0.00001).

**Conclusion:**

The findings in this study suggested that development of the milk ducts in early pregnancy can be observed using ABUS. These findings will be useful to gain a better understanding of breast ultrasound imaging characteristics during pregnancy.

## Introduction

The first transformation of the breast in pregnancy occurs in the first trimester with marked increases in secondary and tertiary ductal branching under the influence of estrogen, providing ductal arbors for the second transformation, alveolar development [1, 2]. de Holanda et al. [2] reported breast ultrasonograms during pregnancy and lactation, but did not provide a detailed description of the first change, i.e., milk duct development on sonography. While there have been few reports on the clinical significance of the changes occurring in early pregnancy, Haku et al. [3] reported that women with marked mammary gland growth from early pregnancy had better0 milk production capacity. However, they also did not characterize the development of milk ducts on sonograms. We postulated that determination of the timing and characteristics of ductal changes first occurring in early pregnancy on ultrasonography would provide a more in-depth understanding of breast ultrasound imaging findings during pregnancy, particularly in the current context of increasing numbers of women delaying childbirth and the high rates of pregnancy-associated breast cancer (PABC) [4, 5]. These findings would also facilitate simpler and more accurate prediction of lactation potential.

Although automated breast ultrasound (ABUS) was originally intended as a complementary imaging technique to mammography for women with dense breast tissue, it has also been used in screening for PABC in Japan as mammography is avoided in pregnant and lactating women in Japan. Therefore, this retrospective study was conducted to characterize the ductal changes occurring in early pregnancy, the timing of their appearance, and their distribution and direction of extension, using images acquired with ABUS.

## Subjects and methods

A total of 357 asymptomatic pregnant women underwent ABUS examinations at Yoyogi Women’s Clinic in Tokyo, Japan, between February 2019 and December 2021. After excluding 1 woman who was currently breastfeeding and 17 whose infants had been weaned within less than 1 year, 339 were included in the analysis.

This study was conducted to determine how and when ductal development in the first trimester becomes evident on ultrasonography. If available, images of pregnant women undergoing ABUS examination before pregnancy and during lactation after childbirth were examined to differentiate between the manifestations of milk duct development in the early stage of pregnancy and normal changes over time.

### ABUS equipment and image reader

ABUS was performed using an ultrasound system that can automatically acquire full-field volumes of the breast in daily practice (Invenia ABUS 2.0; GE Healthcare Systems, Little Chalfont, UK) equipped with a high-frequency reverse curve transducer with a 15-cm field of view and bandwidth of 6–15 MHz [6]. Image acquisition was performed by a nurse or midwife without professional certification from the Japan Society of Ultrasonics in Medicine or the Japanese Central Organization on Quality Assurance of Breast Cancer Screening, who were trained by the manufacturer’s in-house technician. They each had about 1 year of experience before taking images of pregnant women for the analysis. The ABUS examination was carried out with the patient in the supine position. Three views of each breast were taken: lateral, anteroposterior, and medial. The examination took approximately 15 min per patient for both breasts. A 3D data set was generated from these images and sent to a separate workstation, where a radiologist performed the analysis and manipulation of the volume data. All images were evaluated by a single radiologist certified by both the Japan Radiological Society and the Japan Society of Ultrasonics in Medicine, and who had ABUS interpretation experience.

### Criteria for differentiation between pregnant and nonpregnant milk ducts on ultrasonography

**Fig. 1:**
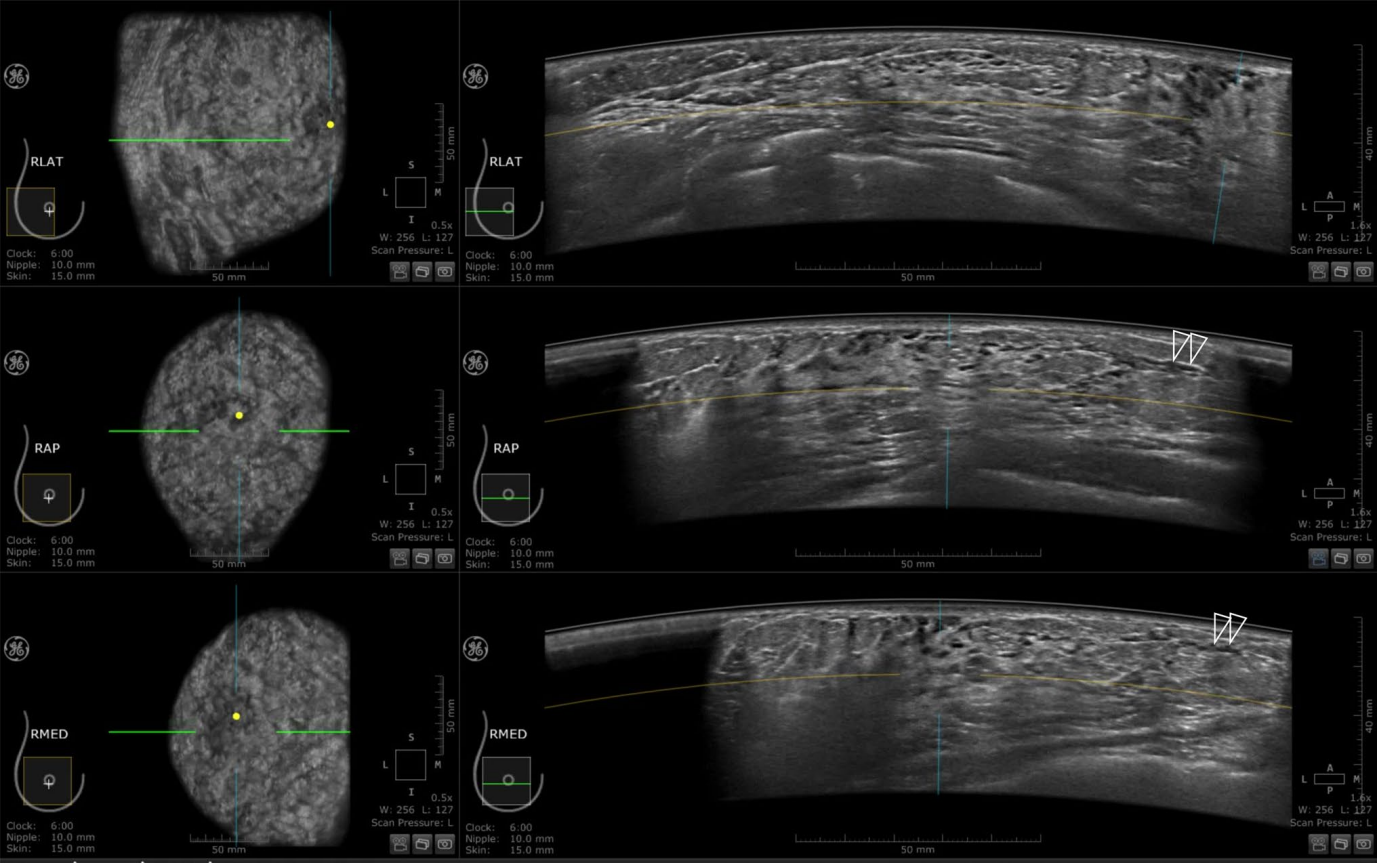
Right breast ABUS images of a 29-year-old woman at 20 weeks 5 days of pregnancy. Lateral (LAT), anteroposterior (AP), and medial (MED) images from top to bottom, with a coronal section presented on the left and a horizontal section on the right. These sonograms show ducts extending to the margins of the breast where little echogenic fibroglandular tissue could be seen (double open arrowheads)

**Fig. 2:**
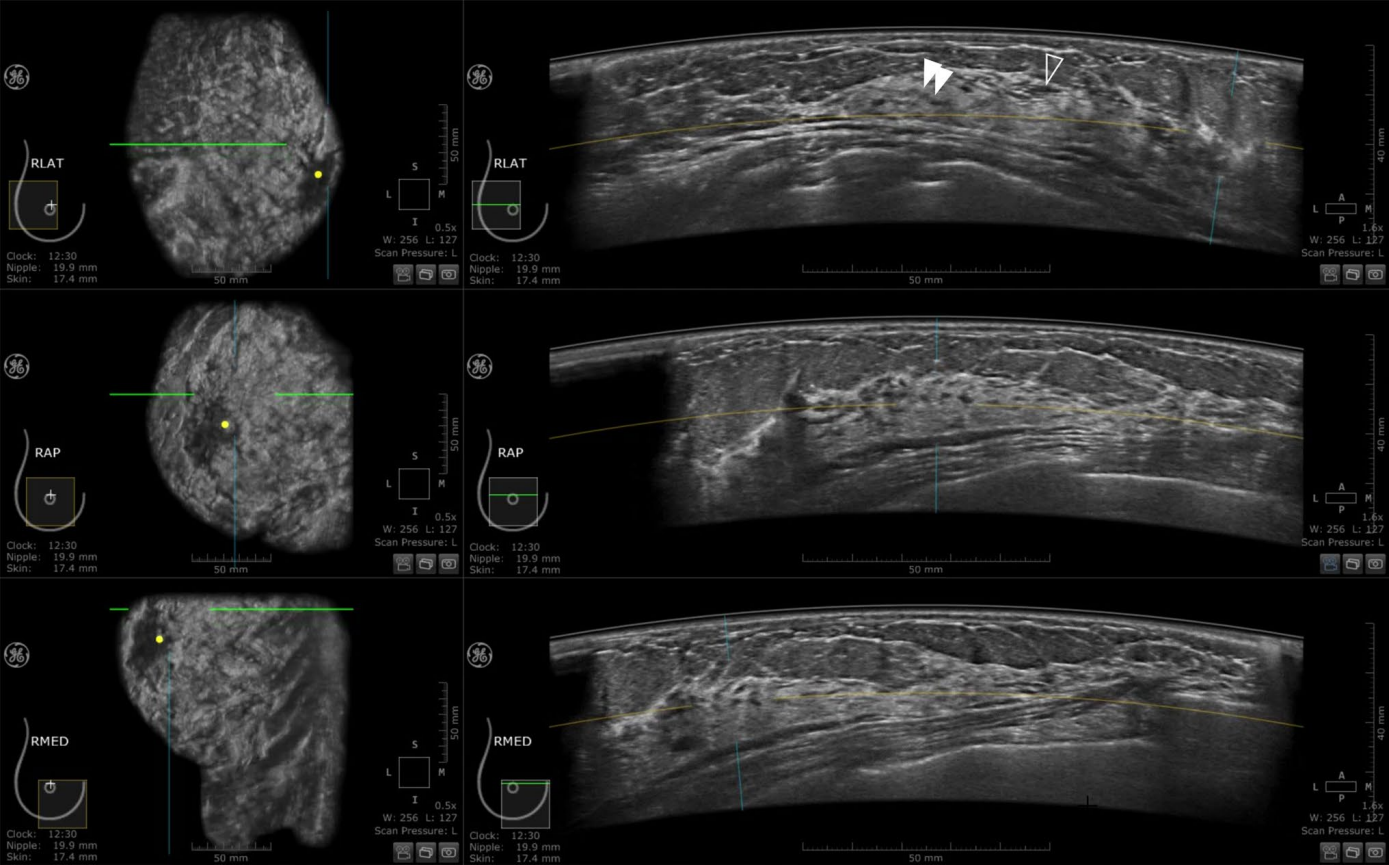
Right breast ABUS images of a 35-year-old woman at 5 weeks 2 days of pregnancy. Lateral (LAT), anteroposterior (AP), and medial (MED) images from top to bottom, with a coronal section presented on the left and a horizontal section on the right. The open arrowhead indicates a ductal image, but it was not sufficiently developed to be considered indicative of the gestational period. Neither criterion 1 nor 2 was met in this case. In addition, the double arrowhead shows an anechoic duct-like structure, which was not determined to be a milk duct but likely corresponded to the stroma supporting the ducts

**Fig. 3:**
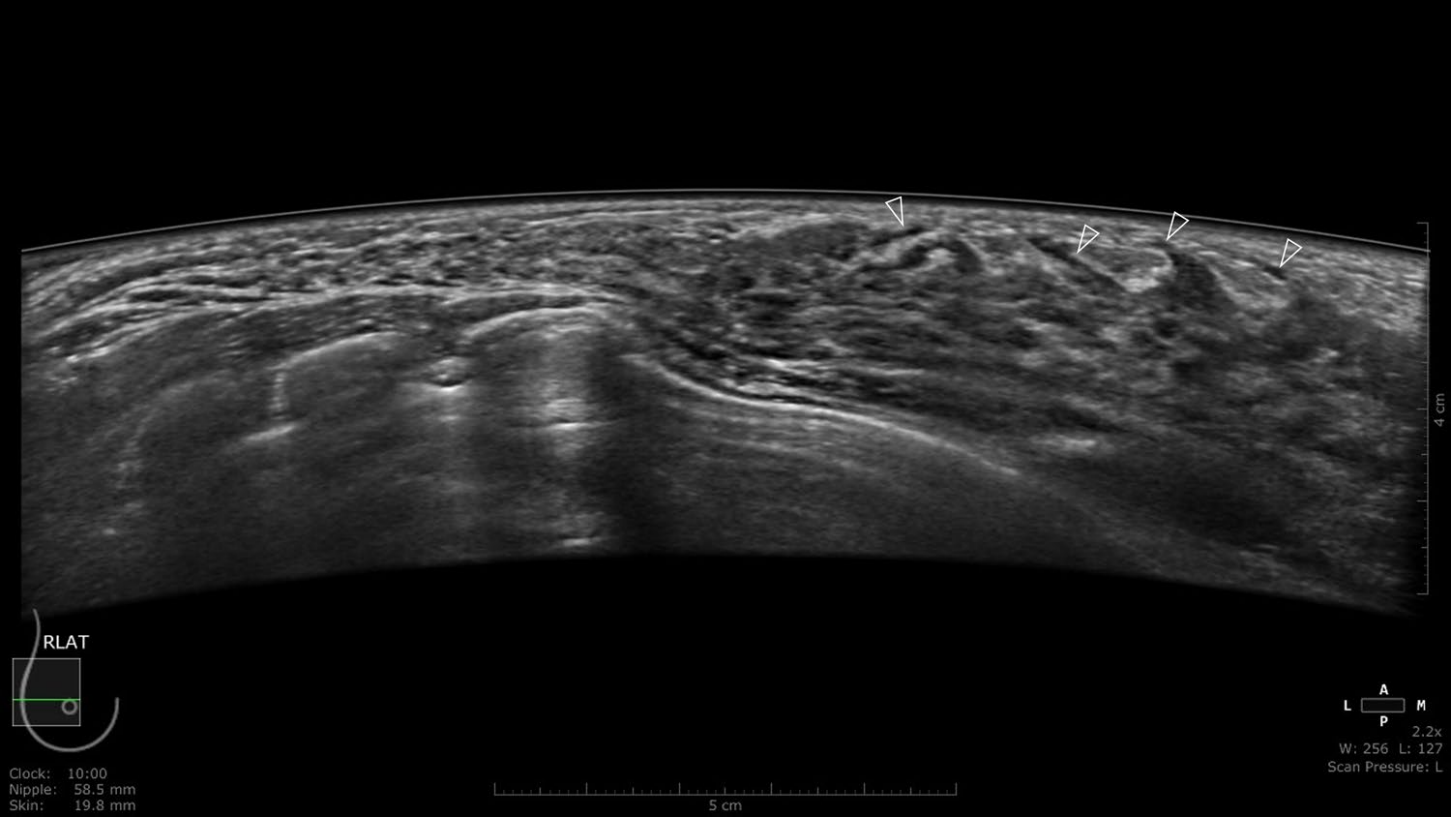
Right breast ABUS images of a 36-year-old woman at 24 weeks 6 days of pregnancy. This image shows ductal structures running along the ascending Cooper’s ligament. As the images were taken in the second trimester, when vascular development is considered also to be apparent, not all can be immediately identified as milk ducts. However, those in which continuity with extramammary ductal structures could not be confirmed were milk ducts

As milk ducts are often observed on ultrasonography even in nonpregnant and nonlactating women, it was difficult to distinguish between the ductal development in early pregnancy and other changes. However, the criteria for changes during pregnancy were set as follows based on the experience of the radiologist:

1. Extension of the ducts to the margins of the breast where there is little or no echogenic fibroglandular tissue on sonography represents the milk duct development occurring in pregnancy (Figs. 1, 2).
2. The appearance of ductal structures running along the ascending Cooper’s ligament tapering off or ending in a blind end at the superficial layer of the superficial fascia represents the milk duct development occurring in pregnancy (Fig. 3).

### Anatomy required for image interpretation

**Fig. 4:**
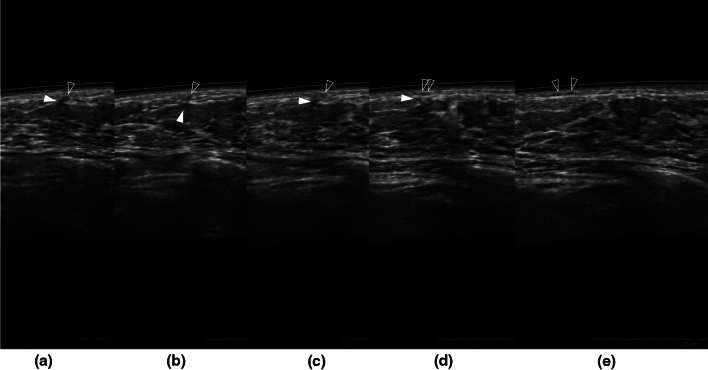
Left breast ABUS images of a 36-year-old woman at 24 weeks 6 days of pregnancy. Images are arranged from left to right in the order **a**–**e**. The ductal structure ran longitudinally across the premammary fascia (**a**) and then penetrated the superficial fascia (**b**, **c**) and ran laterally just below the dermis (**d**, **e**), and therefore, they were vascular structures. The filled arrowhead indicates the deeper structure, and the open arrowhead indicates the shallower structure. However, the deeper vessel is obscure in **e**

As the color Doppler method cannot be used in ABUS, the following points were noted for differentiation of milk ducts from vessels. Milk ducts do not meander and are usually long and linear or segmentally linear along the long axis or elongated oval to circular in shape especially along the short axis, and do not run longitudinally in the fibroglandular region, except for the region of the nipple and subnipple and the exceptions described above along Cooper’s ligament. The milk ducts neither cross the superficial layer nor penetrate the deep layer of the superficial fascia. As it is known to form a vascular network on the superficial layer of the superficial fascia [7], the intramammary ductal structures contiguous with the subdermal ductal structures running horizontally were considered to be blood vessels (Fig. 4a–e). In addition, duct-like structures observed in normal fibroglandular tissue, which were neither anechogenic nor had very low echogenicity, could not be considered as milk ducts due to the possibility of surrounding stroma supporting the ducts (Fig. 2) [8].

In accordance with the above criteria, we investigated the timing of the appearance of changes in the mammary glands of pregnant women. Findings fulfilling either criterion 1 or 2 were taken to indicate a gestational mammary gland. The above criteria were considered hypothetical, and if the rate of positive findings increased with progression of pregnancy, the hypothesis was valid.

### Statistical analysis

Statistical analyses were performed using EZR (Easy R) [9], which is based on R and R Commander (Juichi Medical University Saitama Medical Center, Saitama, Japan). Spearman’s correlation coefficient (*ρ*) was calculated to examine the correlation between the number of weeks of gestation and the prevalence rate of the findings: *ρ* = 0–0.25, no correlation; *ρ* = 0.25–0.50, weak correlation; *ρ* = 0.50–0.75, relatively strong correlation; *ρ* = 0.75–1.0, strong correlation. In all analyses, *P* < 0.05 was taken to indicate statistical significance.

## Results

**Fig. 5:**
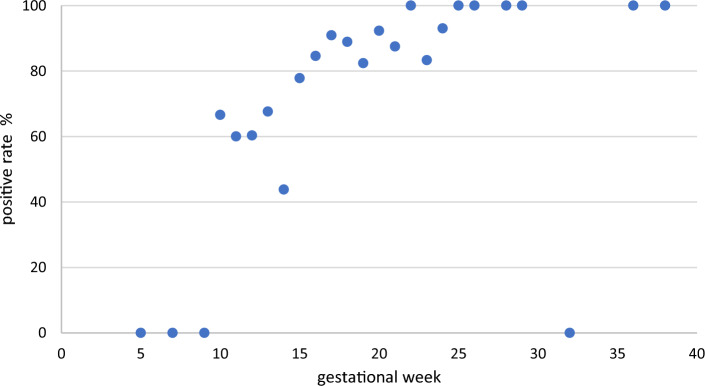
Incidence of the findings by gestational week. With a few exceptions, the rate of occurrence increased from 10 weeks of pregnancy, and then rose gradually to a plateau after 20 weeks

The population consisted of pregnant women ranging in age from 22 to 43 years, with an average age of 33.5 years. The scatterplot in Fig. 5 shows the rate at which ductal development was seen on ultrasound images versus the number of weeks of gestation. With few exceptions, the prevalence rate increased sharply beginning at 10 weeks, and then rose with the progression of gestation, reaching a plateau after 20 weeks (*ρ* = 0.766, *P* < 0.00001). The numbers of pregnant women at each gestational week are shown in Table 1. One pregnant woman at 32 weeks had no evidence of ductal development, and the prevalence of ductal development at 32 weeks was 0%.

**Table 1:** Numbers of pregnant women at each gestational week

Gestational week	5	7	9	10	11	12	13	14	15	16	17	18	19	20	21	22	23	24	25	26	28	29	32	36	38
The number of pregnant	4	2	1	3	10	57	30	16	27	52	11	9	17	39	9	9	6	15	5	5	1	1	1	1	1

The numbers of pregnant women by gestational week correspond to Fig. 5

## Discussion

There was a strong correlation between the number of weeks of gestation and the prevalence of ductal growth findings proposed in this study. Therefore, the presented criteria for development of milk ducts on sonography in pregnancy are likely to be valid. The findings appeared from 10 weeks of gestation in the first trimester, and were consistent with the findings described previously by Macias and Hink [1].

Assuming that our criteria are appropriate, it makes sense that the ducts extend to the margins of the breast, which should result in development of the mammary gland, especially laterally, during lactation. The extension of the ducts along Cooper’s ligament cannot be interpreted easily. However, it suggests that the mammary gland not only undergoes thickening by expansive proliferation but also that the ducts infiltrate the shallow fat layer along the preexisting connective tissue, which may allow the mammary tissue to grow particularly thick.

Even at 20 or more weeks of gestation, 7 of 93 pregnant women showed no evidence of ductal development on sonography. Haku et al. reported that a lack of mammary gland changes during gestation raises concerns about breast milk secretion after delivery, which could be interpreted as a risk of lactation deficiency. However, this was outside the scope of the present study, and as interviews regarding milk secretion are not easy to conduct and require adequate psychological care, further careful studies are required to address this issue.

Although retrospective evaluation of the presence or absence of findings is difficult with conventional ultrasonography, we took advantage of ABUS in this study. However, this study had some limitations. First, the Doppler method cannot be used with ABUS. Second, it was not possible to determine whether these findings could be confirmed via conventional ultrasonography. Finally, the assessments were made by a single radiologist. In future studies, the morphological features for distinguishing between developing milk ducts and blood vessels in early gestation should be verified using high-resolution grayscale ultrasonography and the Doppler method, along with evaluation of reproducibility of the imaging findings.

## Conclusion

Milk ducts extending to the margins of the breast where little or no echogenic fibroglandular tissue can be seen on sonography represent duct development occurring in the gestational period. In addition, milk ducts running along the ascending Cooper’s ligament tapering off and ending in a blind end at the superficial layer of the superficial fascia should represent duct development occurring in the gestational period. The findings in this study suggested that the development of milk ducts in early pregnancy can be observed even using ABUS. These findings will be useful toward gaining a better understanding of breast ultrasound imaging findings in pregnancy.

## Data Availability

The datasets generated and/or analyzed during the current study are available from the corresponding author on reasonable request.
